# Associations of *APOE* Gene Variants rs429358 and rs7412 with Parameters of the Blood Lipid Profile and the Risk of Myocardial Infarction and Death in a White Population of Western Siberia

**DOI:** 10.3390/cimb44040118

**Published:** 2022-04-13

**Authors:** Sergey Semaev, Elena Shakhtshneider, Liliya Shcherbakova, Dinara Ivanoshchuk, Pavel Orlov, Sophia Malyutina, Valery Gafarov, Yuliya Ragino, Mikhail Voevoda

**Affiliations:** 1 Institute of Cytology and Genetics, Siberian Branch of Russian Academy of Sciences (ICG SB RAS), 10 Prospekt Ak. Lavrentyeva, 630090 Novosibirsk, Russia; semaev@bionet.nsc.ru (S.S.); dinara@bionet.nsc.ru (D.I.); orlovpavel86@gmail.com (P.O.); mvoevoda@ya.ru (M.V.); 2 Institute of Internal and Preventive Medicine (IIPM)—Branch of ICG SB RAS, 175/1 Borisa Bogatkova Str., 630089 Novosibirsk, Russia; scherbakovalv@bionet.nsc.ru (L.S.); smalyutina@hotmail.com (S.M.); valery.gafarov@gmail.com (V.G.); ragino@mail.ru (Y.R.)

**Keywords:** rs7412, rs429358, risk of myocardial infarction, risk of death, lipid profile, white population, Western Siberia

## Abstract

The present study aimed to analyze possible associations of rs7412 and rs429358 of the *APOE* gene with lipid profile parameters, the risk of myocardial infarction, and death in the mostly white population of Western Siberia (Russia). The study population was selected from a sample surveyed within the framework of the Health, Alcohol and Psychosocial Factors In Eastern Europe (HAPIEE) study (9360 subjects, age 53.8 ± 7.0 years, males/females 50/50). PCR was conducted with fluorescence detection according to the TaqMan principle on a real-time PCR machine. The frequency of a minor allele (C) of rs429358 was 0.13, and the frequency of a minor allele (T) of rs7412 was 0.09. In our study, the woman with the rare ɛ1/ɛ4 genotype had substantial aberrations in blood lipid levels. In Kaplan–Meier curves, statistically significant differences were revealed in the prognosis of survival within the subgroup of females who had a myocardial infarction (*p* = 0.0006): the prognosis was worse for carriers of the ɛ2/ɛ2 genotype and for ɛ4/ɛ4 carriers. Survival analysis regarding deaths from all causes showed (*p* = 0.0238) that female carriers of the ɛ2/ɛ4 genotype had a worse prognosis than did carriers of other genotypes. Thus, in the population of Western Siberia (Russia), we confirmed statistically significant associations between rs7412 & rs429358 genotypes and lipid profile parameters.

## 1. Introduction

Cardiovascular diseases are the leading cause of death in industrialized countries [1]. Lipid metabolism dysfunction, which underlies the atherosclerotic process, is one of the main risk factors for cardiovascular diseases [2].

Apolipoprotein E (APOE) is a major chylomicron apolipoprotein and is required for the normal catabolism of triglyceride-rich lipoprotein components. The APOE protein is expressed in many cells, primarily in the liver as well as in the brain, spleen, kidneys, sex glands, adrenal glands, and macrophages [3]. The widespread expression of APOE indicates its importance for various processes such as metabolism of lipoproteins, fat-soluble vitamins, and glucose/energy as well as signal transduction, metastasis, and angiogenesis.

The *APOE* gene is located in chromosomal region 19q13.2 and encodes a 317 amino acid (aa) apolipoprotein E precursor (NM_000041.4). After proteolytic removal of the 18 aa signal peptide with subsequent glycosylation, mature APOE is secreted as a 299 aa protein with a relative molecular weight of 34,200 Da.

The variants that give rise to APOE isoforms are rs429358, p.Cys130Arg (ɛ4) and rs7412, p.Arg176Cys (ɛ2). According to the allele frequencies reported in the Genome Aggregation Database (gnomAD), covering the sequencing data from ~100,000 subjects from various disease-specific and population-specific genetic studies, rs429358 minor allele frequency is 0.1425 and rs7412 minor allele frequency is 0.06542 in the total gnomAD population [4]. Approximate frequencies (prevalence rates) of genotypes ɛ2/ε2, ɛ2/ε3, ɛ2/ε4, ɛ3/ε3, ɛ3/ε4, and ɛ4/ε4 in the general population are 0.004, 0.065, 0.009, 0.759, 0.143, and 0.02, respectively [5].

The ε2/ε2 genotype of this gene leads to familial dysbetalipoproteinemia or type III hyperlipoproteinemia, where elevated plasma cholesterol and triglyceride levels are a consequence of impaired clearance of chylomicrons and of remnants of very low-density lipoprotein. Genotypes containing the ε2 allele correlate with lower serum total cholesterol and low-density lipoprotein (LDL-C) cholesterol levels and with higher triglyceride levels as compared to the ε3/ε3 genotype. Genotypes containing the ε4 allele are associated with elevated levels of total cholesterol and LDL-C [5]. Some reports suggest that *APOE* gene polymorphisms can increase the risk of cardiovascular diseases [6].

The present study aimed to analyze possible associations of rs7412 and rs429358 with lipid profile parameters, the risk of myocardial infarction, and death in the mostly white population of Western Siberia (Russia).

## 2. Materials and Methods

### 2.1. Study Population

A cross-sectional epidemiological examination of an adult population was carried out in Novosibirsk (Western Siberia, Russia). The study involved materials from the “Collection of human biomaterials at the Institute of Internal and Preventive Medicine—a branch of ICG SB RAS” (No. 0324-2017-0048). The profile of the group of residents in the surveyed districts is typical for the city of Novosibirsk in terms of ethnicity, age, and employment [7]. From Novosibirsk residents examined within the framework of an international multicenter study on risk factors of cardiovascular diseases in Eastern Europe (HAPIEE; Health, Alcohol, and Psychosocial Factors in Eastern Europe) [7], using a random-number table, a representative population has been chosen previously (9360 subjects, 45–69 years old, age 53.8 ± 7.0 years [mean ± SD], males/females 50/50, whites > 90%). The study protocol was approved by the ethics committee at the Institute of Internal and Preventive Medicine—a branch of the Institute of Cytology and Genetics (ICG), the Siberian Branch of the Russian Academy of Sciences (SB RAS), Novosibirsk, Russia (decision No. 7 of 22 June 2008). From each patient, we obtained informed consent to be examined for the collection and analysis of biological samples.

### 2.2. Measures and Clinical Data

The program of clinical examination included registration of sociodemographic data; a standard questionnaire on smoking and alcohol use; a history of chronic diseases; the use of medications; the Rose cardiological questionnaire; anthropometric data (height, body weight, and waist circumference); three-time measurement of blood pressure; spirometry; electrocardiography; detection of “definite coronary heart disease” according to validated epidemiological (myocardial infarction as determined by electrocardiography, pain-free coronary heart disease according to electrocardiography, or stable effort angina of functional classes II-IV according to the Rose questionnaire) and clinical-functional criteria (according to electrocardiograms interpreted via the Minnesota code); and biochemical assays of blood serum (total cholesterol, high-density lipoprotein cholesterol [HDL-C], triglycerides, and fasting glucose). Blood sampling from the cubital vein was performed in the morning on an empty stomach and at 12 h after a meal. Blood lipid profiling (total cholesterol, triglycerides, HDL-C, and LDL-C) was conducted by enzymatic methods using standard reagents (Biocon Fluitest; Lichtenfels, Germany) on a Labsystem FP-901 biochemical analyzer (Helsinki, Finland). The index of atherogenicity was calculated using the formula: IA = (TC − HDL-C)/HDL-C.

Data collection in the cohort regarding endpoints (myocardial infarction, stroke, and death from all causes) was performed for 7 years from several sources of information: (i) the second clinical examination of the same population in 2013–2015, (ii) a database called the Myocardial Infarction Registry of Novosibirsk City, and (iii) a database called the Stroke Registry of Novosibirsk City.

### 2.3. Genotyping and Quality Control

From the above-mentioned population (*n* = 9360), for molecular genetic analysis, a study population of 2709 subjects was selected by the random number method. Phenol–chloroform extraction was carried out to isolate DNA from the blood samples [8]. The quality of the extracted DNA was assessed using an Agilent 2100 Bioanalyzer capillary electrophoresis system (Agilent Technologies Inc., Santa Clara, CA, USA).

Genotyping of rs429358 and rs7412 (Table 1) was performed by allele-specific real-time PCR with fluorescence detection according to the TaqMan principle (Biolink, Novosibirsk, Russia) on a StepOnePlus Real-Time PCR System (Thermo Fisher Scientific, Foster City, CA, USA). The following oligonucleotide primers and probes (labeled with the FAM dye and the BHQ1 fluorescence quencher) were designed for each SNP under study:

wild-type rs7412: 5′-cctggtacactgccaggcg-3′,

minor allele of rs7412: 5′-cctggtacactgccaggca-3′,

common primer for rs7412: 5′-gccagagcaccgaggagct-3′,

probe for rs7412: 5′-[FAM]agcttgcgcaggtgggaggc[BHQ1]-3′,

wild-type rs429358: 5′-cggacatggaggacgtgt-3′,

minor allele of rs429358: 5′-cggacatggaggacgtgc-3′,

common primer for rs429358: 5′-gctctggccgagcatgg-3′,

probe for rs429358: 5′-[FAM]cggccgcctggtgcagt[BHQ1]-3′.

Each of the four RT-PCRs was carried out in a 25 μL volume, which included 15 ng of DNA, 2 μL of primer mixture “wild type” or “minor allele” for rs7412 or primer mixture “wild type” or “minor allele” for rs429358, 5 μL of the master mix (Biolink, Novosibirsk, Russia), and 16 μL of water. The thermal cycling program was as follows: initial denaturation for 180 s at 95 °C, followed by 5 cycles of 10 s at 95 °C and 60 s at 60 °C and then 35 cycles of 10 s at 95 °C and 50 s at 60 °C.

Laboratory personnel performing the genotyping assays were blinded to the physical- and clinical-examination data.

Rare genotypes (ɛ1/ɛ2, ɛ1/ɛ4, and ɛ2/ɛ2) and some of the common variants were additionally verified by direct automated Sanger sequencing.

### 2.4. Statistical Analyses

These analyses of the data were carried out using the statistical software package SPSS for Windows. The significance level was set to 0.01. To assess the data on poor long-term prognosis of cardiovascular diseases, the method of survival tables and log-rank test were employed, and the results were graphically visualized by the Kaplan–Meier analysis [9].

The significance of differences in allele frequencies in the studied groups and compliance with the Hardy–Weinberg equilibrium were evaluated by the χ^2^ test. Differences in the means of continuous variables between genotype groups were adjusted for sex, age, and body–mass index via the GLM model in the SPSS software for Windows.

## 3. Results and Discussion

The main characteristics of the study population are presented in Table 2. The ratio of males to females was 47:53.

### 3.1. Frequencies of Alleles and Genotypes of rs429358 & rs7412 (the APOE Gene)

For rs429358 and rs7412 in the study population (*n* = 2709), the distribution of genotypes’ frequencies was found to comply with the Hardy–Weinberg equilibrium (χ^2^ = 0.68 and χ^2^ = 0.54, respectively). The frequency of a minor allele (C) of rs429358 was 0.13, and the frequency of a minor allele (T) of rs7412 was 0.09 in the mostly white population of Western Siberia, consistently with the distribution of allele frequencies in white populations of Eastern and Western Europe, according to gnomAD—Genomes European: for rs429358 C, the frequency is 0.14 (https://gnomad.broadinstitute.org/variant/19-45411941-T-C?dataset=gnomad_r2_1 accessed on 4 April 2022), for rs7412 T, the frequency is 0.07 (https://gnomad.broadinstitute.org/variant/19-45412079-C-T?dataset=gnomad_r2_1 accessed on 4 April 2022).

In our study population (from Western Siberia), the ε3 allele is the most common: frequency 0.7763 in the male subgroup, 0.7832 in the female subgroup, and 0.7799 for both sexes (Table 3). The results are consistent with data on the frequency of the ɛ3 allele worldwide [6,10,11]. Frequencies of alleles ε2 and ε4 are comparable with the distribution of frequencies in white populations of Eastern and Western Europe [12,13].

Carriers of rare genotypes ɛ1/ɛ2 and ɛ1/ɛ4 were found among the examined individuals. In the literature, there are three rare isoforms of apolipoprotein E that have been identified by isoelectric focusing: ɛ1, ɛ5, and ɛ7 [14]. As compared to ɛ3, variant ɛ1 (CM890010) has less than 10% of binding activity toward the LDL receptor [15]. In Russia, there is a clinical case of familial dysbetalipoproteinemia with the carriage of the *APOE* ɛ1/ɛ2 genotype in a patient with atherosclerosis (early development), acute coronary syndrome, elevated levels of LDL-C and triglycerides, and hyperglycemia [16]. In our study, the male carrier of the ɛ1/ɛ2 genotype (age 61; body–mass index 31.5) showed slight aberrations of blood lipid levels [total cholesterol 213.4 mg/dL (5.52 mmol/L), LDL-C 123.7 mg/dL (3.2 mmol/L), HDL-C 49.1 mg/dL (1.27 mmol/L), triglycerides 88.38 mg/dL (1.01 mmol/L), atherogenicity index 3.37, and no chylomicronemia]. The patient’s questionnaire showed the absence of heart attacks and cerebrovascular pathology both in the patient himself and in his relatives. Ɛ1/ɛ2 genotype carriage is a necessary but not sufficient condition for the development of dyslipidemia. According to the literature, hyperglycemia makes a significant contribution to the development of dyslipidemia in carriers of the ɛ2 variant [17]. Because the ɛ1/ɛ2 genotype carriage was detected here in a patient within a populational study, an obvious limitation is the lack of genetic and biochemical data from the patient’s relatives.

In our study, the woman with the rare ɛ1/ɛ4 genotype (age 63; body–mass index 27.94) had substantial aberrations in blood lipid levels: total cholesterol 369.59 mg/dL (9.56 mmol/L), LDL-C 224.23 mg/dL (5.8 mmol/L), HDL-C 57.99 mg/dL (1.50 mmol/L), triglycerides 437.5 (5.00 mmol/L), atherogenicity index 5.36, and no chylomicronemia. The patient’s questionnaire revealed the absence of heart attacks and cerebrovascular pathology both in the patient herself and in her relatives. Nonetheless, during the observation period, the patient suffered an attack of acute coronary syndrome in 2009 (at the age of 64 years) and an ischemic stroke in 2012 (at age 67). Our findings imply the role of the ε4 variant as a major factor influencing blood lipid levels and increasing the risk of cardiovascular diseases.

Chronologically, ε4 is believed to be the ancestral allele from which variants ε3 and ε2 sequentially evolved more than 200,000 years ago [18]. According to the literature, in comparison with other variants, in ε4 carriers, apolipoprotein has a distinct binding affinity for lipoprotein particles or LDL receptors. Apolipoprotein ε4 has higher binding activity for triglyceride-rich lipoproteins (such as chylomicron remnants and very-low-density lipoproteins) than for LDLs. This property of ε4 leads to a decrease in LDL-C clearance, resulting in higher levels of LDL-C as compared to carriers of *APOE* ε2 and ε3 [19]. According to another classic hypothesis, the elevated level of LDLs in carriers of the ɛ4 allele is due to underexpression of LDL receptors because of accelerated absorption (by the liver) of very-low-density lipoproteins enriched in apolipoprotein ɛ4 [5]. The ɛ4 allele is an established risk factor for aging and various age-related diseases such as several types of dementia (including Alzheimer’s disease) and cardiovascular diseases [20]. In a meta-analysis of studies on various populations (all relevant reports and references from original and review articles published from 1966 to January 2004: 15,492 cases and 32,965 controls), it was demonstrated that ɛ3/ɛ4 carriers and ɛ4/ɛ4 carriers have a 1.4-fold higher risk of coronary heart disease [21].

### 3.2. Associations of rs429358 & rs7412 with Parameters of the Blood Lipid Profile

We next analyzed these associations with total cholesterol, triglycerides, HDL-C, LDL-C, and the atherogenicity index. The highest total-cholesterol levels (mean values) were found in male carriers of the ε4 allele. Among females, high total cholesterol levels were found in carriers of the ε4 allele and ε2/ε2 homozygotes.

Differences between genotypes in mean LDL-C and triglyceride levels and in the mean atherogenicity index were statistically significant both in the study population (*p* < 0.0001) and in subgroups: males (*p* < 0.0001 for LDL-C, *p* = 0.001 for triglycerides, and *p* = 0.003 for the atherogenicity index) and females (*p* < 0.0001 for LDL-C, *p* < 0.0001 for triglycerides, and *p* < 0.0001 for the atherogenicity index). Differences in total cholesterol levels among *APOE* genotypes were statistically significant in the study population (*p* < 0.0001) and in the female subgroup (*p* < 0.0001; Table 4).

Thus, in the population of Western Siberia (Russia), we confirmed statistically significant associations between rs7412 & rs429358 genotypes and triglycerides, LDL-C, the atherogenicity index (in the study population and both sex subgroups), and total cholesterol (in the study population and female subgroup).

### 3.3. Survival Analysis for Carriers of Common Apolipoprotein E Isoforms

This analysis for common APOE isoforms (ɛ2/ɛ4, ɛ2/ɛ2, ɛ2/ɛ3, ɛ3/ɛ3, ɛ3/ɛ4, and ɛ4/ɛ4) in the study population and separately in the male and female subgroups was carried out by the Kaplan–Meier method [9,22]. The endpoints were myocardial infarction, stroke, and all-cause mortality.

In the Kaplan–Meier curves, statistically significant differences were revealed in the prognosis of survival within the subgroup of females who had a myocardial infarction (*p* = 0.0006): the prognosis was worse for carriers of the ɛ2/ɛ2 genotype and for ɛ4/ɛ4 carriers. In the subgroup of males who had a myocardial infarction (Figure S1) and in the whole study population (Figure S2), there were no statistically significant differences in the survival prognosis among *APOE* genotypes (*p* = 0.6819 and *p* = 0.3209, respectively).

Survival analysis regarding deaths from all causes showed (*p* = 0.0238) that female carriers of the ɛ2/ɛ4 genotype had a worse prognosis than did carriers of other genotypes (Figure S3).

In the study population, the survival analysis regarding deaths from all causes uncovered statistically significant differences between some genotypes (*p* = 0.0262) (Figure S4). Namely, carriers of the ɛ2/ɛ4 genotype had a worse prognosis too. In the male subgroup, there were no statistically significant differences among genotypes in the prognosis of survival in terms of deaths from all causes (*p* = 0.5297) (Figure S5).

There were no statistically significant differences among carriers of different genotypes in the analysis of survival after stroke either in the study population (Figure S6) or among males (Figure S7) and females (Figure S8) separately (*p* = 0.3339, 0.4965, and 0.9132, respectively).

**Figure 1:**
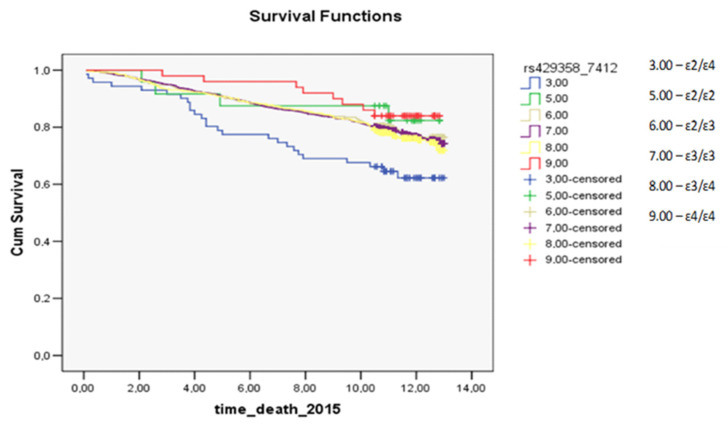
The survival plot for myocardial infarction in the female subgroup: the prognosis was worse for carriers of the ɛ2/ɛ2 genotype (green curve) and for ɛ4/ɛ4 carriers (red curve) (*p* = 0.0006). time_death_2015: this is the time (in years) from the start of the observation (December 2002) to the end of the study (the occurrence of the outcome or the end of the observation period: December 2015). Cum Survival: the probability that the case has no outcome until the time point that we choose along the horizontal axis.

In the analysis of survival after myocardial infarction, a statistically significant difference among carriers of dissimilar genotypes was observed in the female subgroup (*p* = 0.0006; Figure 1): the prognosis was worse for carriers of the ɛ2/ɛ2 genotype and for ɛ4/ɛ4 carriers. For deaths from all causes, differences in the prognosis of survival were statistically significant between some genotypes in the female subgroup and the study population (*p* = 0.0238 and *p* = 0.0262, respectively, the prognosis was worse for carriers of the ɛ2/ɛ4 genotype). The survival plots for all groups regarding all-cause mortality, myocardial infarction, and stroke are presented in the Supplementary Materials.

### 3.4. Discussion

When analyzing the association of rs7412 and rs429358 with lipid profile parameters in the mostly white population of Western Siberia (Russia), we found the highest mean levels of total cholesterol in male carriers of the ɛ4 allele, in line with data from other studies [5]. Among females, a high mean level of total cholesterol was documented here both in carriers of the ɛ4 allele and in ɛ2 homozygotes. Judging by the results of major global studies involving UK Biobank data, genotypes ε3/ε4 and ε4/ε4 are associated with hypercholesterolemia and coronary heart disease in contrast to the ε3/ε3 genotype, whereas the ε2/ε3 genotype has shown a protective effect against these pathologies [6]. In a research article by Rassmussen K.L. et al. [23], carriage of the ɛ2 allele in a heterozygous state correlated with a moderate decrease in the concentration of total blood cholesterol and with elevated levels of remnant cholesterol. In ɛ2 homozygotes, in the presence of other risk factors, type III hyperlipidemia (familial dysbetalipoproteinemia) is reported to develop [5]. Besides, according to the literature, ɛ2 homozygosity is associated with lowered levels of LDL-C and elevated concentrations of triglycerides as compared to other genotypes [17]. In our study, the lowest mean level of LDL-C was registered in the subgroup of males with the ɛ2/ɛ2 genotype (*p* < 0.0001); however, in the female subgroup and the study population, the lowest levels of LDL-C were seen in individuals with the ɛ2/ɛ3 genotype. The association of ɛ2 homozygosity with an increased level of triglycerides in comparison with other genotypes in our study is in agreement with international data in all groups (*p* = 0.001 for males, *p* < 0.0001 in the study population and the female subgroup) [5]. In our study, the rare ɛ1/ɛ4 genotype turned out to be the most unfavorable in terms of hypercholesterolemia risk in females.

**Figure 2:**
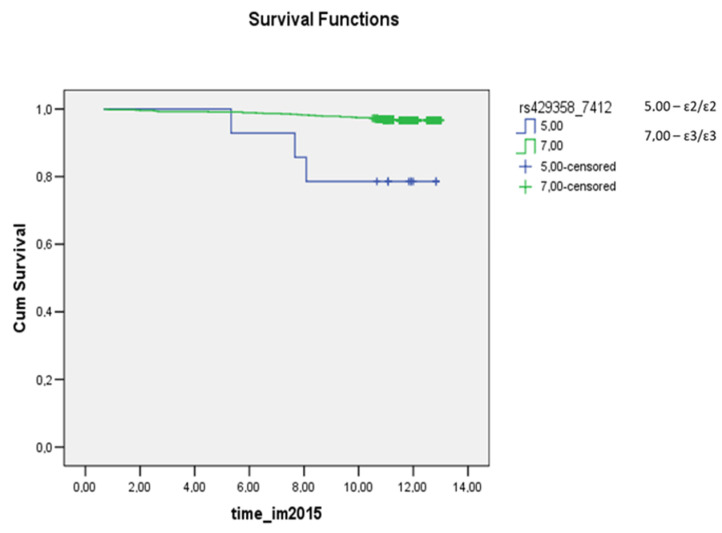
The survival plot for myocardial infarction in the female subgroup. The prognosis was worse for ɛ2/ɛ2 carriers (blue curve) versus the widespread ɛ3/ɛ3 genotype (green curve) (*p* < 0.0001). time_im2015: this is the time (in years) from the start of the observation (December 2002) to the end of the study (the occurrence of myocardial infarction or the end of the observation period: December 2015). Cum Survival: the probability that the case has no outcome until the time point that we choose along the horizontal axis.

In another work, involving data from the UK Biobank (345,659 whites without a history of coronary heart disease, mean age 56.5 years, 45.7% males), the highest survival rate regarding deaths from all causes was observed in carriers of the ε2 allele, whereas ε4 carriers had the worst prognosis [19]. In a cohort of 9241 people, Frikke-Shmidt R. et al. tested the hypothesis that the risk of coronary heart disease depends on the *APOE* genotype in women and men [24]. According to their findings, the ε2/ε3 genotype in women has a protective effect relative to the ε3/ε3 genotype, whereas men with genotypes ε3/ε4 and ε4/ε4 are at a higher risk of coronary heart disease. In our female subgroup, homozygous carriage of the ε4 allele correlated with a worse prognosis regarding survival after myocardial infarction, but no protective effect of the ε2 allele was detectable. The prognosis of survival after myocardial infarction was worse for homozygous carriers of the ε2 allele among females (*p* < 0.0001; Figure 2).

In a study on 7418 people (age 56.7 ± 12.4 years [mean ± SD], 68% of males) from the Secondary Manifestations of ARTerial disease project (SMART: a prospective cohort study on patients with cardiovascular disease or cardiovascular risk factors), Koopal C. et al. [17] found no association between the *APOE* genotype and coronary heart disease, stroke, or cardiovascular mortality. Our work revealed a statistically significant association between various genotypes and survival after myocardial infarction in the female subgroup (*p* = 0.0006): the prognosis was worse for carriers of genotypes ɛ2/ɛ2 and ɛ4/ɛ4. In the female subgroup and the study population, some differences in the prognosis of survival regarding deaths from all causes were statistically significant too (*p* = 0.0238 and *p* = 0.0262, respectively): the prognosis was worse for the ɛ2/ɛ4 genotype carriers.

In contrast to some previous studies [25,26,27], we did not notice a protective effect of the ε2 allele in our study population in terms of either mean levels of total cholesterol or survival after myocardial infarction. On the other hand, our findings on the ɛ4 allele are consistent with the data from other authors [5,6,20,28].

### 3.5. Limitations

We analyzed only rs429358 and rs7412 (the *APOE* gene) and traditional cardiovascular risk factors and therefore could not exclude the influence of other factors that may affect observational studies. Biochemical blood assays in our patients did not include remnant cholesterol (intermediate-density and very-low-density lipoprotein cholesterol). Only one male (ɛ1/ɛ2 genotype) and one female (ɛ1/ɛ4 genotype) are carriers of rare variants in our study.

### 3.6. Conclusions

Investigation into genetic risk factors of cardiovascular diseases in various populations is important not only for the analysis of disease outcomes but also for the selection of primary prevention groups by taking into account human genetic diversity. Patients at high genetic risk may get additional motivation to adhere to a healthy lifestyle.

## Figures and Tables

**Table 1:** Variants of the *APOE* gene.

rs429358	rs7412	rs429358 & rs7412 Genotype	ID for rs429358 & rs7412 Genotype in Survival Plots
(C;C)	(T;T)	ɛ1/ɛ1	1
(C;T)	(T;T)	ɛ1/ɛ2	2
(C;T)	(C;T)	ɛ2/ɛ4	3
(C;C)	(C;T)	ɛ1/ɛ4	4
(T;T)	(T;T)	ɛ2/ɛ2	5
(T;T)	(C;T)	ɛ2/ɛ3	6
(T;T)	(C;C)	ɛ3/ɛ3	7
(C;T)	(C;C)	ɛ3/ɛ4	8
(C;C)	(C;C)	ɛ4/ɛ4	9

**Table 2:** Baseline characteristics of the study population (*n* = 2709, Western Siberia, Russia) were randomly selected for molecular genetic analysis.

	Males	Females	Both Sexes
Number of subjects, n	1263	1446	2709
Age, years	56.7 ± 0.2	56.6 ± 0.2	56.6 ± 0.1
TC, mg/dL	241.5 ± 1.4	259.2 ± 1.5	250.9 ± 1.1
HDL-C, mg/dL	58.3 ± 0.4	61.4 ± 0.5	59.9 ± 0.3
LDL-C, mg/dL	119.5 ± 1.3	132.4 ± 1.3	126.4 ± 0.9
TGs, mg/dL	141.6 ± 2.2	144.8 ± 2.2	143.3 ± 1.6
Index of atherogenicity	3.4 ± 0.04	3.4 ± 0.04	3.4 ± 0.03
Body–mass index, kg/m^2^	27.2 ± 0.1	30.3 ± 0.2	28.8 ± 0.1

Continuous variables are presented as mean ± standard error. Abbreviations: HDL-C, high-density lipoprotein cholesterol; LDL-C, low-density lipoprotein cholesterol; TC, total cholesterol; TGs, triglycerides.

**Table 3:** Frequencies of alleles and genotypes of rs429358 & rs7412 in the study population.

	Among Males	Among Females	In Both Sexes
	Frequency, Number of Subjects	Frequency, Number of Subjects	Frequency, Number of Subjects
Genotypes			
ɛ1/ɛ1	- *n* = 0	- *n* = 0	- *n* = 0
ɛ1/ɛ2	0.001 *n* = 1	- *n* = 0	0.0005 *n* = 1
ɛ2/ɛ4	0.032 *n* = 40	0.021 *n* = 31	0.026 *n* = 71
ɛ1/ɛ4	- *n* = 0	0.001 *n* = 1	0.0005 *n* = 1
ɛ2/ɛ2	0.008 *n* = 10	0.010 *n* = 14	0.009 *n* = 24
ɛ2/ɛ3	0.144 *n* = 182	0.127 *n* = 183	0.135 *n* = 365
ɛ3/ɛ3	0.610 *n* = 771	0.618 *n* = 893	0.614 *n* = 1664
ɛ3/ɛ4	0.188 *n* = 237	0.205 *n* = 296	0.197 *n* = 533
ɛ4/ɛ4	0.017 *n* = 22	0.019 *n* = 28	0.018 *n* = 50
Allele frequencies			
ε1	0.0004	0.0004	0.0004
ε2	0.0962	0.0837	0.0895
ε3	0.7763	0.7832	0.7799
ε4	0.1271	0.1328	0.1301

**Table 4:** Associations of rs429358 & rs7412 genotypes with parameters of the blood lipid profile in the mostly white population of Western Siberia (*n* = 2709).

Sex	Genotype	TC, mg/dL	HDL-C, mg/dL	LDL-C, mg/dL	TGs, mg/dL	Index of Atherogenicity
Males	ɛ1/ɛ1	-	-	-	-	-
	ɛ1/ɛ2	206.9 ± 49.2	52.6 ± 14.8	124.2 ± 43.8	67.0 ± 72.7	3.0 ± 1.3
	ɛ2/ɛ4	254.3 ± 7.8	56.1 ± 2.4	119.7 ± 6.9	174.4 ± 11.5	3.8 ± 0.2
	ɛ1/ɛ4	-	-	-	-	-
	ɛ2/ɛ2	227.9 ± 15.6	52.3 ± 4.7	93.5 ± 13.9	182.7 ± 23.0	3.5 ± 0.4
	ɛ2/ɛ3	230.4 ± 3.7	58.3 ± 1.1	103.2 ± 3.3	153.3 ± 5.4	3.2 ± 0.1
	ɛ3/ɛ3	241.3 ± 1.8	59.2 ± 0.5	121.1 ± 1.6	135.5 ± 2.6	3.3 ± 0.1
	ɛ3/ɛ4	244.6 ± 3.2	56.3 ± 1.0	125.7 ± 2.9	139.1 ± 4.8	3.6 ± 0.1
	ɛ4/ɛ4	245.0 ± 10.5	55.3 ± 3.2	125.9 ± 9.4	141.8 ± 15.5	3.6 ± 0.3
*p*		0.033	0.100	<0.0001 *	0.001 *	0.003 *
Females	ɛ1/ɛ1	-	-	-	-	-
	ɛ1/ɛ2	-	-	-	-	-
	ɛ2/ɛ4	254.0 ± 9.9	60.0 ± 3.5	127.7 ± 8.9	137.7 ± 14.4	3.4 ± 0.3
	ɛ1/ɛ4	359.6 ± 55.2	58.0 ± 19.2	214.3 ± 48.9	194.0 ± 78.9	5.3 ± 1.4
	ɛ2/ɛ2	319.8 ± 14.7	58.3 ± 5.2	142.8 ± 13.1	264.0 ± 21.1	4.7 ± 0.4
	ɛ2/ɛ3	245.0 ± 4.1	63.9 ± 1.4	114.7 ± 3.6	147.7 ± 5.9	3.1 ± 0.1
	ɛ3/ɛ3	258.2 ± 1.9	61.6 ± 0.7	133.6 ± 1.7	139.6 ± 2.7	3.4 ± 0.1
	ɛ3/ɛ4	264.5 ± 3.2	59.5 ± 1.1	138.5 ± 2.9	148.0 ± 4.6	3.7 ± 0.1
	ɛ4/ɛ4	255.1 ± 10.6	63.1 ± 1.7	128.8 ± 9.4	140.3 ± 15.2	3.3 ± 0.3
*p*		<0.0001 *	0.346	<0.0001 *	<0.0001 *	<0.0001 *
Both sexes	ɛ1/ɛ1	-	-	-	-	-
	ɛ1/ɛ2	215.8 ± 52.9	53.4 ± 17.4	130.4 ± 46.7	70.9 ± 76.5	3.1 ± 1.3
	ɛ2/ɛ4	255.3 ± 6.3	58.3 ± 2.1	124.1 ± 5.6	158.2 ± 9.1	3.6 ± 0.2
	ɛ1/ɛ4	356.8 ± 52.9	55.5 ± 17.4	212.0 ± 46.7	198.8 ± 76.5	5.4 ± 1.3
	ɛ2/ɛ2	281.3 ± 10.8	55.2 ± 3.6	122.0 ± 9.5	231.4 ± 15.6	4.3 ± 0.3
	ɛ2/ɛ3	237.8 ± 2.8	61.3 ± 0.9	109.0 ± 2.5	150.0 ± 4.0	3.1 ± 0.1
	ɛ3/ɛ3	250.3 ± 1.3	60.5 ± 0.4	127.7 ± 1.2	137.7 ± 1.9	3.4 ± 0.03
	ɛ3/ɛ4	255.5 ± 2.3	58.0 ± 0.8	132.7 ± 2.0	144.2 ± 3.3	3.6 ± 0.1
	ɛ4/ɛ4	251.7 ± 7.6	59.1 ± 2.5	128.2 ± 6.7	143.0 ± 10.9	3.5 ± 0.2
*p*		<0.0001 *	0.071	<0.0001 *	<0.0001 *	<0.0001 *

The data are presented as mean ± standard error. * Statistically significant.

## Data Availability

The data presented in this study are available on request from the corresponding author. The data are not publicly available due to privacy concerns.

## Supplementary Materials

The following supporting information can be downloaded at https://www.mdpi.com/article/10.3390/cimb44040118/s1: Figure S1: The survival plot for stroke in the study population, Figure S2: The survival plot for stroke in the male subgroup, Figure S3: The survival plot for stroke in the female subgroup, Figure S4: The survival plot for myocardial infarction in the study population, Figure S5: The survival plot for myocardial infarction in the male subgroup, Figure S6: The survival plot for all-cause mortality in the study population, Figure S7: The survival plot for all-cause mortality in the male subgroup, Figure S8: The survival plot for all-cause mortality in the female subgroup.

## Abbreviations

HDL-C	high-density lipoprotein cholesterol
LDL-C	low-density lipoprotein cholesterol
PCR	polymerase chain reaction
TC	total cholesterol
TGs	triglycerides
