# Supplementary material for: Associations of APOE Gene Variants rs429358 and rs7412 with Parameters of the Blood Lipid Profile and the Risk of Myocardial Infarction and Death in a White Population of Western Siberia
Source: Curr Issues Mol Biol. 2022 Apr 13;44(4):1713–24. doi: 10.3390/cimb44040118 (PMC9164079; doi:10.3390/cimb44040118)
Supplement: Supplementary file 1 [file cimb-44-00118-s001.zip › cimb-1670512-supplementary.pdf]

Supplementary material

The survival plots for all groups regarding all-cause mortality, myocardial infarction, and stroke are presented in this document.

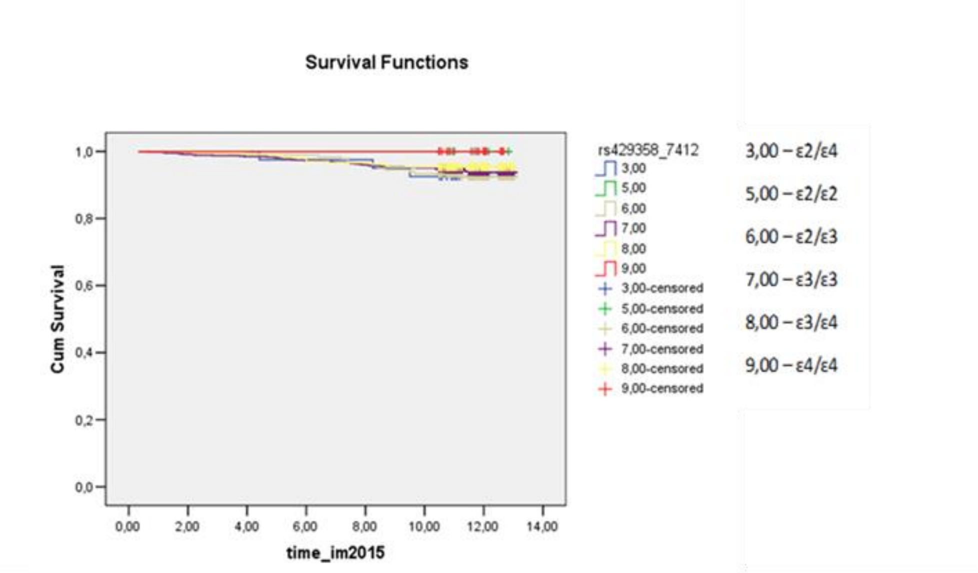

Figure S1. The survival plot for myocardial infarction in the male subgroup.

There were no statistically significant differences in survival prognosis among *APOE* genotypes (p = 0.6891; Figure S1). time_im2015: this is the time (in years) from the start of the observation (December 2002) to the end of the study (the occurrence of a myocardial infarction or the end of the observation period: December 2015). Cum Survival: the probability that the case has no outcome until the time point that we choose along the horizontal axis.

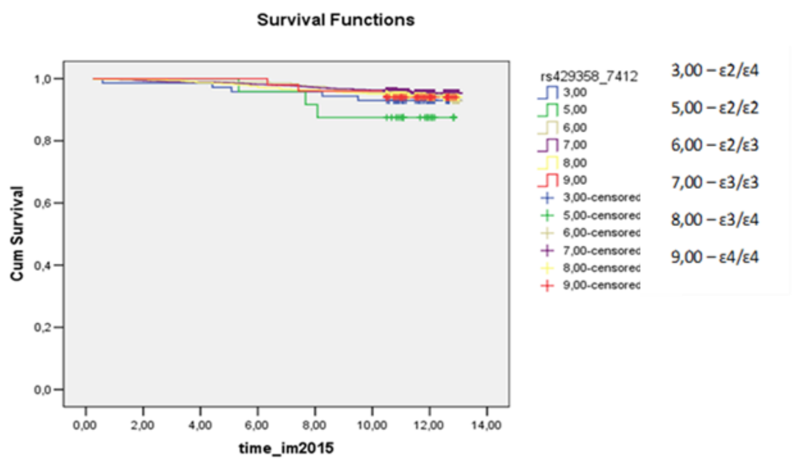

Figure S2. The survival plot for myocardial infarction in the study population.

There were no statistically significant differences in survival prognosis among *APOE* genotypes (p = 0.3209; Figure S2). time_im2015: this is the time (in years) from the start of the observation (December 2002) to the end of the study (the occurrence of a myocardial infarction or the end of the observation period: December 2015). Cum Survival: the probability that the case has no outcome until the time point that we choose along the horizontal axis.

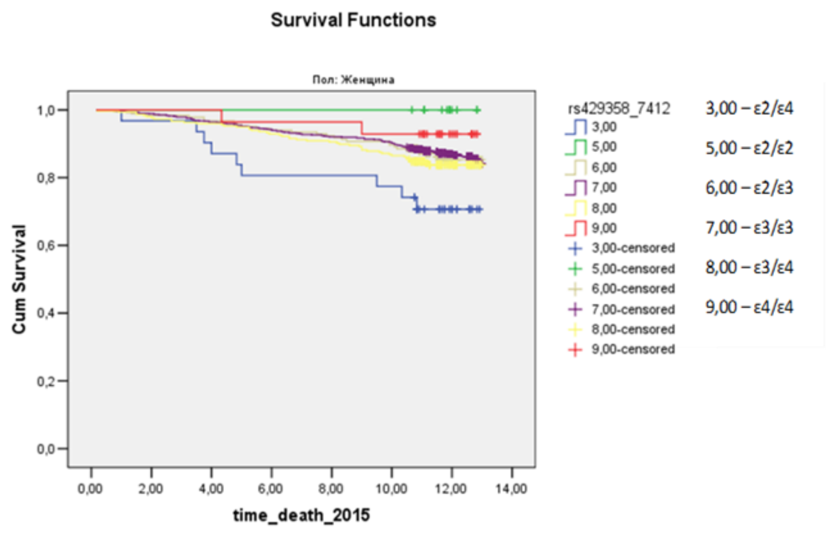

Figure S3. The survival plot for all-cause mortality in the female subgroup.

Female carriers of the ε2/ε4 genotype had a worse prognosis than did carriers of other genotypes ($p = 0.0238$; Figure S3). time_death_2015: this is the time (in years) from the start of the observation (December 2002) to the end of the study (the occurrence of the outcome or the end of the observation period: December 2015). Cum Survival: the probability that the case has no outcome until the time point that we choose along the horizontal axis.

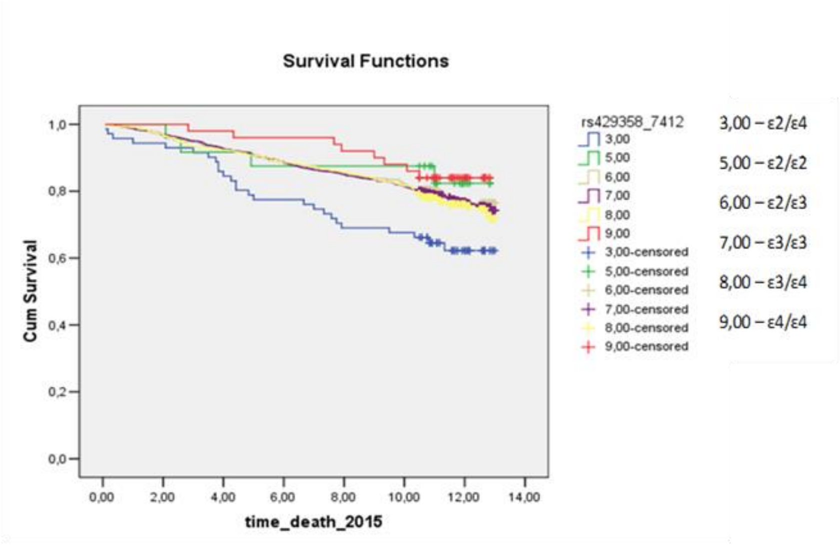

Figure S4. The survival plot for all-cause mortality in the study population.

There were statistically significant differences in the survival prognosis among APOE genotypes ($p = 0.0262$; Figure S4). time_death_2015: this is the time (in years) from the start of the observation (December 2002) to the end of the study (the occurrence of the outcome or the end of the observation period: December 2015). Cum Survival: the probability that the case has no outcome until the time point that we choose along the horizontal axis.

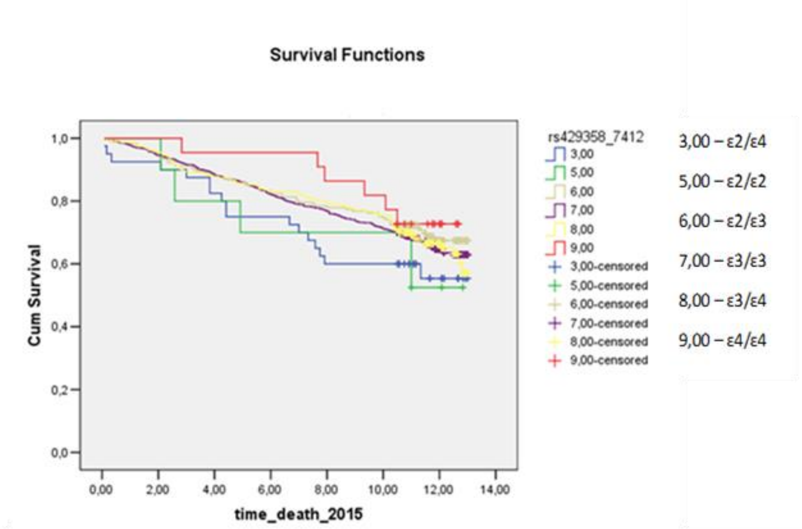

Figure S5. The survival plot for all-cause mortality in the male subgroup.

There were no statistically significant differences among genotypes in the prognosis of survival in terms of deaths from all causes (p = 0.5297; Figure S5). time_death_2015: this is the time (in years) from the start of the observation (December 2002) to the end of the study (the occurrence of the outcome or the end of the observation period: December 2015). Cum Survival: the probability that the case has no outcome until the time point that we choose along the horizontal axis.

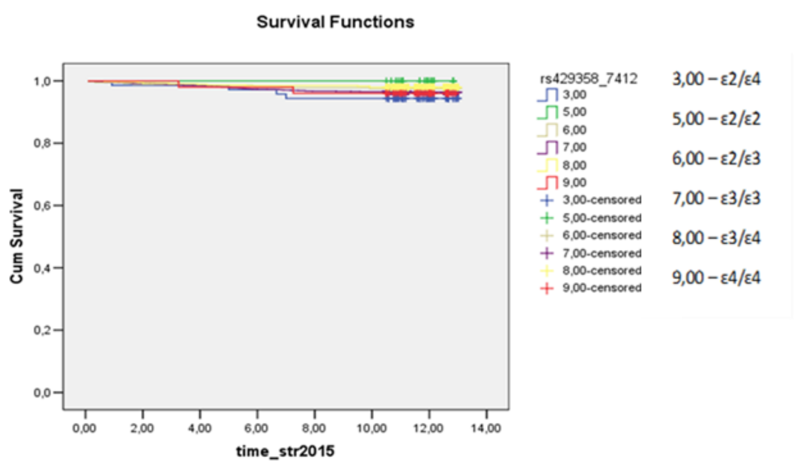

Figure S6. The survival plot for stroke in the study population.

There were no statistically significant differences among carriers of different genotypes (p = 0.3339; Figure S6). time_str2015: this is the time (in years) from the start of the observation (December 2002) to the end of the study (the occurrence of stroke or the end of the observation period: December 2015). Cum Survival: the probability that the case has no outcome until the time point that we choose along the horizontal axis.

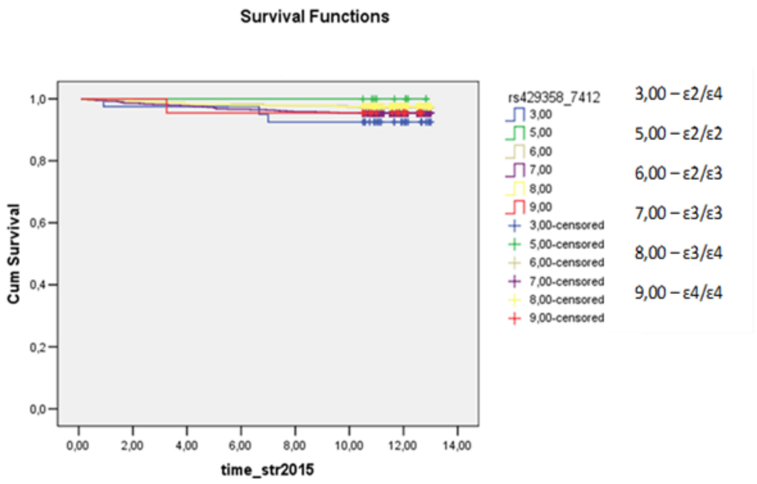

Figure S7. The survival plot for stroke in the male subgroup.

There were no statistically significant differences among carriers of different genotypes (p = 0.4965; Figure S7). time_str2015: this is the time (in years) from the start of the observation (December 2002) to the end of the study (the occurrence of stroke or the end of the observation period: December 2015). Cum Survival: the probability that the case has no outcome until the time point that we choose along the horizontal axis.

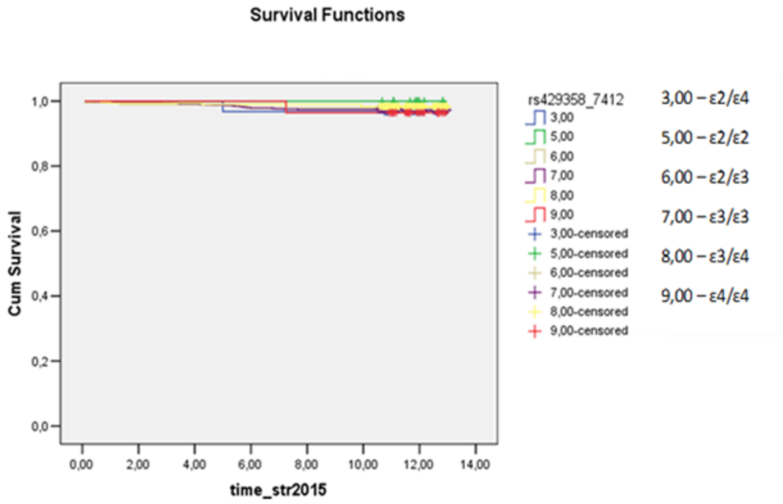

Figure S8. The survival plot for stroke in the female subgroup.

There were no statistically significant differences among carriers of different genotypes (p = 0.9132; Figure S8). time_str2015: this is the time (in years) from the start of the observation (December 2002) to the end of the study (the occurrence of stroke or the end of the observation period: December 2015). Cum Survival: the probability that the case has no outcome until the time point that we choose along the horizontal axis.
